# Interrogation of the human cortical peptidome uncovers cell-type specific signatures of cognitive resilience against Alzheimer’s disease

**DOI:** 10.1038/s41598-024-57104-z

**Published:** 2024-03-26

**Authors:** G. R. Morgan, B. C. Carlyle

**Affiliations:** 1 Department of Physiology, Anatomy & Genetics, University of Oxford, Oxford, OX1 3QU UK; 2 Kavli Institute for Nanoscience Discovery, University of Oxford, Oxford, OX1 3QU UK

**Keywords:** Neuroscience, Alzheimer's disease, Neurodegeneration

## Abstract

Alzheimer’s disease (AD) is characterised by age-related cognitive decline. Brain accumulation of amyloid-β plaques and tau tangles is required for a neuropathological AD diagnosis, yet up to one-third of AD-pathology positive community-dwelling elderly adults experience no symptoms of cognitive decline during life. Conversely, some exhibit chronic cognitive impairment in absence of measurable neuropathology, prompting interest into cognitive resilience—retained cognition despite significant neuropathology—and cognitive frailty—impaired cognition despite low neuropathology. Synapse loss is widespread within the AD-dementia, but not AD-resilient, brain. Recent evidence points towards critical roles for synaptic proteins, such as neurosecretory VGF, in cognitive resilience. However, VGF and related proteins often signal as peptide derivatives. Here, nontryptic peptidomic mass spectrometry was performed on 102 post-mortem cortical samples from individuals across cognitive and neuropathological spectra. Neuropeptide signalling proteoforms derived from VGF, somatostatin (SST) and protachykinin-1 (TAC1) showed higher abundance in AD-resilient than AD-dementia brain, whereas signalling proteoforms of cholecystokinin (CCK) and chromogranin (CHG) A/B and multiple cytoskeletal molecules were enriched in frail vs control brain. Integrating our data with publicly available single nuclear RNA sequencing (snRNA-seq) showed enrichment of cognition-related genes in defined cell-types with established links to cognitive resilience, including SST interneurons and excitatory intratelencephalic cells.

## Introduction

The number of adults diagnosed with dementia is anticipated to triple globally by 2050, the leading cause of which is Alzheimer’s disease (AD)^1^. Brain presence of extracellular amyloid-β (Aβ) deposits and intracellular tau aggregations are regarded as prerequisites for an AD diagnosis. Yet, up to one third of community-dwelling older adults that display significant AD-like neuropathology post-mortem experience negligible reported cognitive decline in life^2,3^. In fact, just ~ 41% of the variance in cognitive trajectory between individuals is ascribable to brain presence of Aβ, tau or other neuropathologies^4–6^. The diverse individual susceptibility to cognitive decline in the presence or absence of AD pathology has inspired the terms cognitive ‘resilience’ and ‘frailty’. As such, resilient individuals retain cognitive fitness in the face of marked Aβ and tau deposition, whereas frail individuals experience chronic cognitive decline despite lack of AD or other gross neuropathology on autopsy. Unravelling the mechanisms that lead to resilience and frailty may provide new insight into therapeutics that improve patient livelihood, independent of brain pathology status.

Whilst current understanding of cognitive resilience and frailty is limited, synaptic function and dendritic spine morphology are likely crucial. Synapse loss is typical in the AD-dementia brain, and is correlated to cognitive impairment, whereas synaptic terminal integrity is preserved to the levels of the healthy brain in AD-resilient subjects^7–9^. Several proteins have been linked to cognitive resilience, many of which are synapse related. VGF (non-acronymic), a neurosecretory presynaptic protein, is consistently downregulated in AD brain and cerebrospinal fluid (CSF), and increased VGF expression is associated with better cognitive outcomes in AD^6,10–15^. Other synaptic signalling molecules positively associated with cognitive fitness in AD include neuritin-1 (NRN1)^11–13,16^ and brain-derived neurotrophic factor (BDNF)^17,18^, whereas proteins associated with cognitive frailty include gut-derived cholecystokinin (CCK) and the chromogranins A/B (CHGA/B)^11,19^. Recent studies have linked cognitive resilience with the survival of particular cell types, including subsets of somatostatin (SST) interneurons (INs) and excitatory intratelencephalic (IT) pyramidal cells^14,20,21^. Identifying which neurons govern resilience mechanisms in AD may offer important insight towards targeted future therapies.

**Figure 1:**
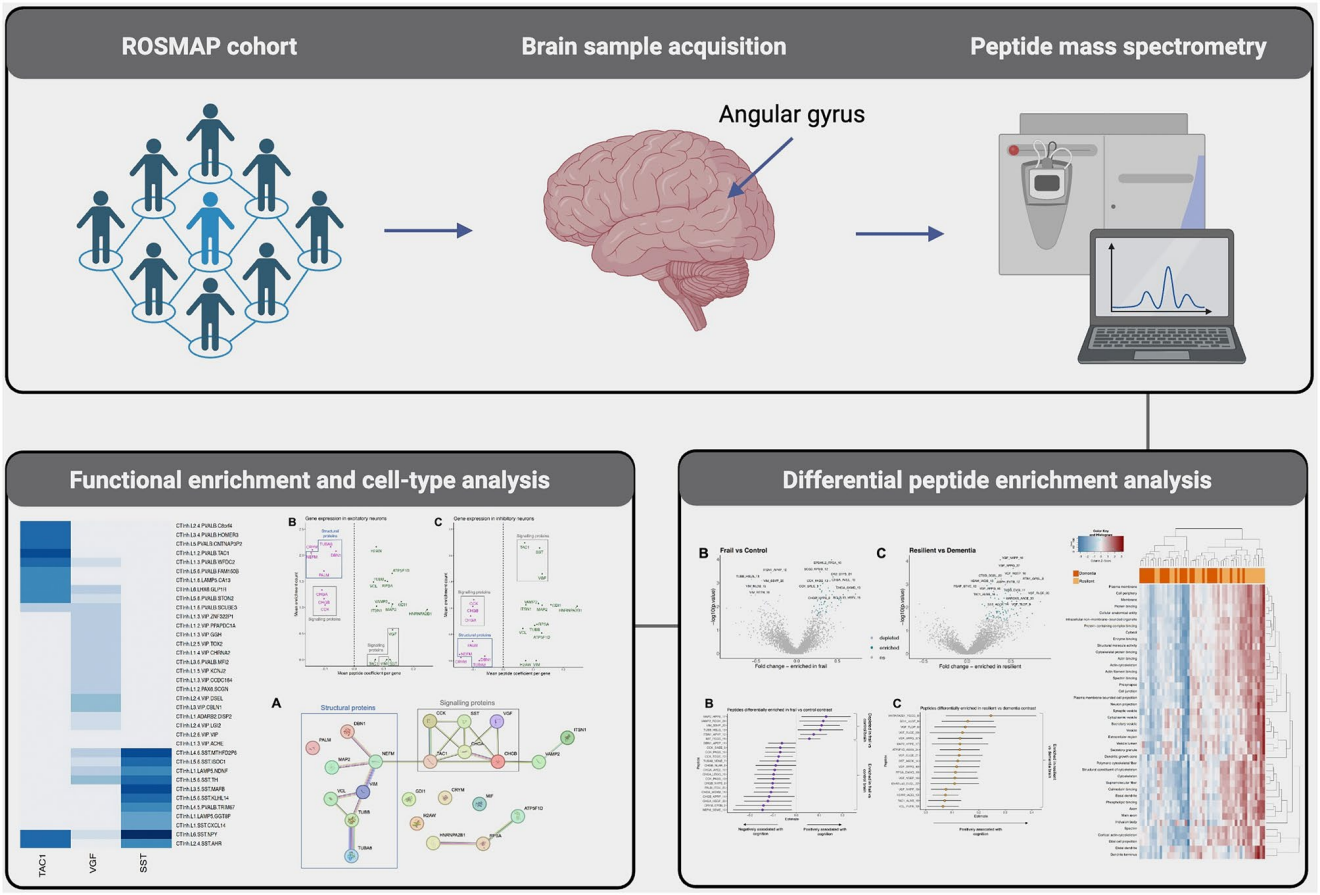
Simplified workflow of the current study. Angular gyrus samples were obtained from 102 participants of the Religious Orders Study and Memory and Aging Projects (ROSMAP). Participants were grouped into four diagnostic classes: control, resilient, frail and dementia. Samples were analysed by peptidomic mass spectrometry (MS). Data were quality controlled and analysed for differential peptide enrichment. Functionally enriched biological pathways were explored and integrated with a single nucleus sequencing database, to define cell types with particular relevance to AD-related cognitive trajectories. Created with BioRender.com.

Critically, many synaptic signalling proteins function via their peptide derivatives. Interrogating brain tissue at the proteomic level may therefore oversimplify a complex picture, neglecting subtle alterations within the peptidome. Thus, to further our knowledge of mechanisms underlying resilience and frailty, the current study (Fig. 1) used nontryptic (peptidomic) liquid chromatography mass spectrometry (LC–MS) on 102 post-mortem cortical samples derived from individuals in four distinct diagnostic classes: control (low neuropathology, retained cognition), AD-resilient (high neuropathology, retained cognition), frail (low neuropathology, impaired cognition) and AD-dementia (high neuropathology, impaired cognition). Brain tissue was collected from the angular gyrus of the parietal association cortex (Brodmann Area 39); an area with widespread connectivity and established roles in higher cognitive function, including memory^22–24^. Further, the angular gyrus was selected due to the susceptibility of this region to neuropathology during intermediate Braak stages of AD^25,26^. 39 proteoforms deriving from 25 parent genes were significantly associated with cognitive resilience and frailty, including proteoforms of VGF, SST, CCK and CHGA/B. Integrating our data with existing single-nuclear sequencing information, we found subpopulations of cortical SST INs that express signalling genes linked to cognitive resilience, whilst glutamatergic neurons were enriched for cytoskeletal genes associated with frailty^14,20^. Our data strengthen current interest surrounding defined neuronal subpopulations in varying cognitive trajectories of older adults^14,20^.

## Methods

### Participants

Post-mortem brain tissue samples were obtained from 102 participants from the Rush Religious Orders Study and Memory and Aging projects, together regarded as ROSMAP^27^. Tissue was collected from the angular gyrus of the parietal association cortex (Brodmann Area 39) due to the susceptibility of this region to Aβ and tau neuropathology during intermediate Braak stages of AD, allowing separation of participants along a clinical spectrum^25,26^. The ROS and MAP longitudinal studies entail annual cognitive and clinical testing of participants, including medical history collection, neurological evaluation and a battery of 19 cognitive tests known to accurately characterise cognitive decline in older adults^28,29^. Scores from annual testing were converted to Z-scores as per the cohort baseline mean (> 1700 participants). To obtain a global cognition Z-score, participants were annually assessed on 19 tests evaluating five cognitive domains: episodic memory, semantic memory, working memory, perceptual orientation, and perceptual speed. The global cognition score is weighted across the 19-test battery assessing these domains. The global cognition Z-score used for the current study is the final valid Z-score collected before death. The global pathology Z-score represents a quantitative summary of Aβ (neuritic and diffuse plaque) and tau burden across five brain regions: the midfrontal cortex, midtemporal cortex, inferior parietal cortex, entorhinal cortex, and hippocampus. All participants provided written informed consent for brain donation upon death. Tissue was prepared for proteomic analysis under an Exempt Secondary Use protocol approved by the Massachusetts General Hospital Institutional Review Board (2016P001074) and all experiments were performed in accordance with relevant guidelines.

**Figure 2:**
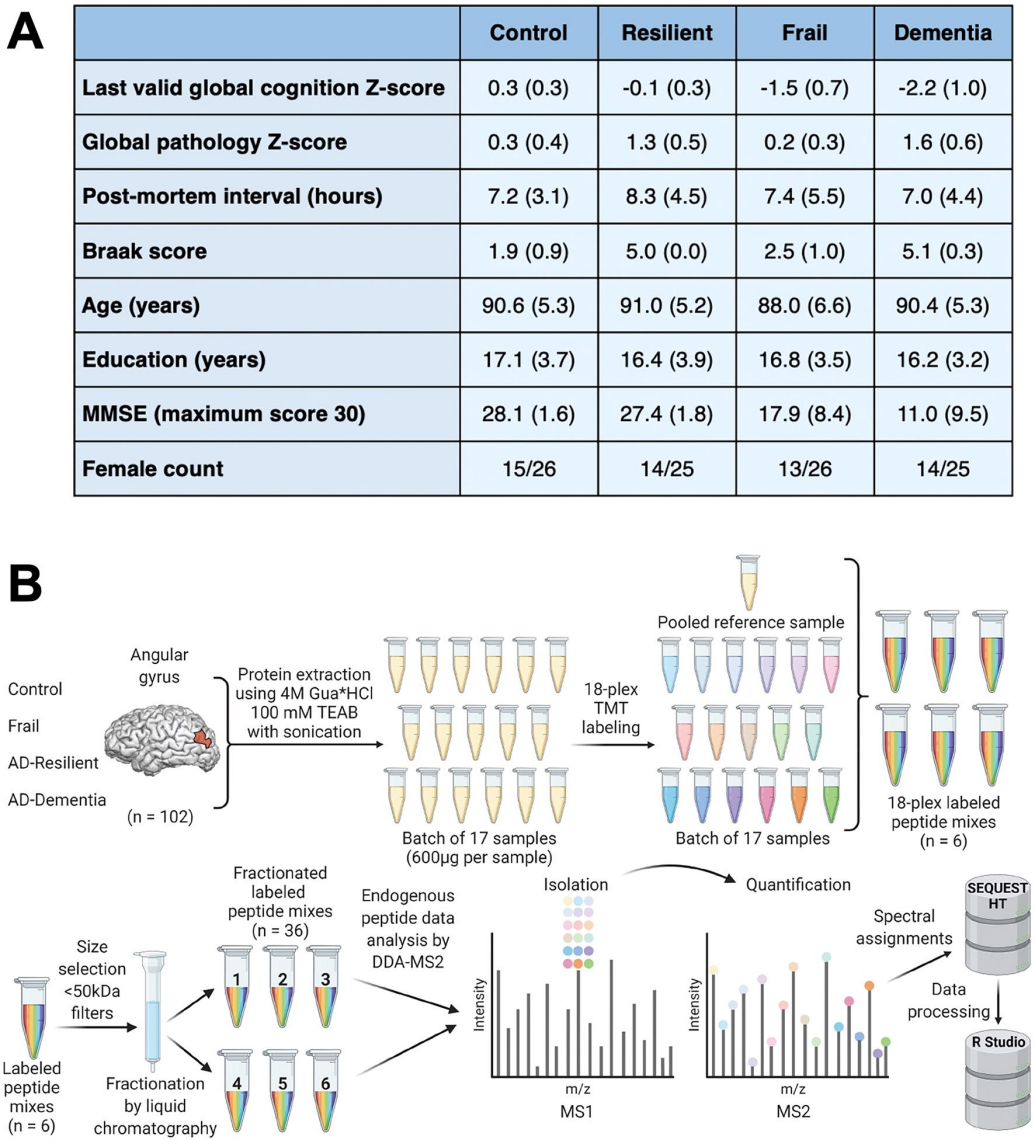
Case demographic summary and representation of mass spectrometry (MS) methods. (**A**) Summary demographics of cases. Data is presented as mean (standard deviation). Last valid global cognition Z-score is a ROSMAP cohort-wide Z-score normalised summary value encompassing a battery of cognitive tests assessing 5 cognitive domains at the last valid clinical visit prior to death. Global pathology Z-score is a quantitative summary score of amyloid and tau pathology burden averaged across 5 brain regions presented as a ROSMAP cohort-wide Z-score. MMSE = Mini Mental State Examination. (**B**) MS methods for the quantification of brain proteoforms from 102 participants within control, frail, resilient and dementia diagnostic classes. Protein was extracted and samples were tandem mass tag (TMT) labelled alongside a pooled reference sample. Samples were mixed and size-selected, for peptide enrichment, via a 50 kDa molecular weight filter. Offline fractionation was employed, resulting in six analytical fractions per plex, each of which underwent data-dependent acquisition MS. Data were collected and analysed at the MS2 level. MS schematic taken from Quinn et al.^19^.

As previously defined^11,19^, a Braak score of ≤ 4 was regarded as ‘low’ pathology, whereas a Braak score > 4 indicated ‘high’ brain pathology^26^. Participants were categorised as cognitively impaired/unimpaired via longitudinal clinical consensus. From these metrics, participants were stratified into four clinical groups: neuropathological AD diagnosis with dementia (“dementia”, 25 participants), neuropathological AD diagnosis without dementia (“resilient”, 25 participants), no AD or other neurological disease diagnosis with dementia (“frail”, 26 participants), and a control group with no AD or other neurological disease diagnosis and negligible cognitive decline at time of death (“control”, 26 participants). Individuals classed as frail exhibited no difference in non-Aβ/tau neuropathological signatures when compared to the other groups (Lewy body, vascular or TAR DNA binding protein 43 (TDP-43) pathology). Participants were well matched across groups in terms of mean age, sex, education level and post-mortem interval (Fig. 2A).

### Brain tissue sample preparation for mass spectrometry

The mass spectrometry experiments have been introduced previously^19^ (Fig. 2B). Briefly, human post-mortem brain tissue was lysed by sonication in 4 M Guanidine hydrochloride in 100 mM TEAB. Samples, including a reference pool, were TMT labelled and pooled into six analytical TMTpro™ 18 plexes of approximately 9 mg of protein per plex. The pooled samples were processed through 50 kDa ultrafiltration cartridges (Amicon) to enrich for endogenous peptides. The flow-through was reduced with TCEP, alkylated with iodoacetamide and treated with hydroxylamine. Samples were purified by solid phase extraction using OASIS HLB (Vac RC 30 mg, Waters). Bound peptides were washed with 5% ACN, 0.1% TFA and eluted with 50% ACN, 0.1% TFA. The eluate was split into two parts; 10% for labelling efficiency and equimolarity checks and 90% for analysis. Samples for analysis were fractionated via Pierce High pH Reversed-Phase Peptide Fractionation kit (Thermo Fisher Scientific) according to Manufacturer’s instructions. Fractions 1 & 2 and 7 & 8 were combined to produce 6 fractions which were dried via SpeedVac and stored at − 80 °C.

### Mass spectrometry data acquisition

Samples were analysed using the EASY-nLCTM 1000 system coupled to an Orbitrap FusionTM TribridTM Mass Spectrometer (both Thermo Scientific) applying a semi-targeted data-dependent acquisition method with an inclusion list for the peptide targets of interest. The list of the targets of interest was generated according to results obtained in a previous study focussed on granin family peptides^19^. Although the inclusion list targets were prioritised for fragmentation in each duty cycle, a lower priority scan event employed a regular data-dependent acquisition scheme to achieve broad coverage of the brain peptidome.

Re-suspended peptides were loaded onto a nanoViper C18 Acclaim PepMap 100 trap column (PN 164946, Thermo Scientific) and resolved using an increasing gradient of ACN in 0.1% Formic acid through a 50 cm 75 um ID EasySpray analytical column (PN ES803A, Thermo Scientific) at a flow rate of 200 nL/min during the first 160 min and 300 nL/min over the last 20 min. The LC gradient started at 5% ACN and increased linearly up to 40% ACN over the first 160 min, then 90% ACN was held constant over the last 20 min. Full scans were acquired at 120.000 resolution with a 3 s duty cycle, fragment scans were acquired at 50.000 resolution, with an automatic gain control (AGC) target of 5e4. Peptide precursors were isolated in a 1.2 Th wide isolation window and fragmented by higher collision-induced dissociation (HCD) at a normalized collision energy of 32%.

### Computational mass spectrometry

In total, 36 separate raw mass spectrometry data files (6 plexes with 6 fractions each) were searched in Proteome Discoverer (PD) v2.5 (Thermo Scientific) using the SEQUEST HT Search algorithm. The raw spectra were searched against a UniprotKB reviewed human database (version January 2021), with no enforced enzyme specificity. TMTpro modification of N-termini and lysine residues, as well as carbamidomethylation of cysteine were set as static modifications; but no variable modifications were allowed due to the already expanded search space of the non-enzymatic search. The precursor mass tolerance was set to 20 ppm, while the fragment mass tolerance was set to 0.02 Da. The false discovery rate was controlled at 1% on PSM level by the Percolator node incorporated in Proteome Discoverer. The reporter ions quantifier node was set up to extract the raw intensity values for TMTpro 18plex mono-isotopic ions. All raw reporter ion intensity values were exported to tab delimited text files for further processing and bioinformatic analysis. The reporter ion intensities of all unmatched spectra with an average reporter ion signal to noise ratio above 10 were also exported and used for input normalization of the individual channels.

Data pre-processing was performed in Proteome Sciences proprietary DIANA software. All data integration tools were developed to work with TMT labelled MS data^30,31^ and include functionality for dealing with isolation interference^32^, isotopic crosstalk^33^, Peptide Spectral Match (PSM) normalisation and summarisation into peptides. Filtering of PSMs was conducted using isolation interference information from the input Proteome Discoverer multi-consensus file. The threshold of 50% was applied to guarantee that at least half of the signal comes from the peptide itself. Isotope impurity correction was applied to PSM level data to address impurities due to isotopic overlap of the different reporter ion masses. Isotope correction factors used in this procedure were specific to the production batch of TMTpro reagents used for labelling. Then, ratios of reporter ion intensities were calculated for experimental samples relative to the reference sample and log2-transformed. Data belonging to identical peptide sequences were median summarised to transform the PSM data matrix into a peptide matrix. Reporter ion intensities were median scaled, and ratios of reporter ion intensities were calculated for the experimental samples relative to the pooled sample.

Prior to QC filtering, 22,314 peptides were identified at the MS2 stage, 86% of which were present in more than one sample. Any peptide with > 20% values missing was excluded from further analysis, leaving 3696 peptides in the final dataset. Missing values were not imputed. Peptide notation is reported as the gene name, the first four amino acid residues, and the length of the peptide, e.g., CHGA_LEGQ_18.

### Data analysis

In our previous publication, the analysis of this dataset only considered six genes/proteins; VGF, secretogranin (SCG) 1, 2, 3 & 5, and CHGA^19^. In this paper, we present the first proteome-wide analysis of this dataset. All analysis and figure creation was carried out in R Studio. Data were analysed and plotted using the *tidyverse*, *tibble, broom, ggplot2, gplots, ggrepel, heatmap.2, patchwork, UpSetR,* and *RColorBrewer* packages. Firstly, 3696 parallel linear models were constructed, re-setting the reference factor to compare all possible permutations of diagnostic class, with peptide quantification as outcome variables and categorical demographic information as explanatory variables:

$$\begin{aligned} & Peptide \, quantification \, ratio\sim categorical \, diagnostic \, class\left( {control, \, resilient, \, frail, \, dementia} \right) \, \\ & \quad + \,age \, at \, death + sex + postmortem \, interval + education \, years \\ \end{aligned}$$

Secondly, 3696 parallel linear models were constructed with peptide quantification as outcome variables, and continuous demographic information as explanatory variables:

$$\begin{aligned} & Peptide \, quantification \, ratio\sim global \, cognition \, Z score + global \, pathology \, Z score \, \\ & \quad + age \, at \, death + sex + postmortem \, interval + education \, years \\ \end{aligned}$$

This similar second model was constructed to allow consideration of nuanced interparticipant differences that may be lost by stratifying individuals into discrete classes. Benjamini Hochberg p-value adjustment was performed to adjust for multiple testing. An alpha level of 0.05 for adjusted p-values was considered statistically significant.

Significant peptides were searched for biological pathway enrichment and parent protein interactions using the STRING database (Version 12)^34^. A background set of 11,744 brain-expressed proteins taken from a recent large-scale proteomic study was used for these analyses^13^. Human brain single-nucleus sequencing data was obtained^35^ and compared to our peptide enrichment data, to assess which cell types may underlie peptide changes. Normalised mean gene enrichment scores (raw gene expression/mean expression of protein of interest across all cell types) for grouped excitatory and inhibitory neuronal types were plotted against the coefficients from the continuous linear regression output for significant peptides. Analysis was performed at the gene level. All code used for the current analysis and figure creation, alongside copies of supplementary figures, can be found here.

## Results

### Association of peptide proteoforms with age, sex and post-mortem interval

Nontryptic mass spectrometry was performed on 102 human cortical samples obtained post-mortem from the angular gyri of individuals stratified into four diagnostic classes: neuropathological AD diagnosis with dementia (“dementia”, 25 participants), neuropathological AD diagnosis without dementia (“resilient”, 25 participants), no AD or other neurological disease diagnosis with dementia (“frail”, 26 participants), and a control group with no AD or other neurological disease diagnosis and negligible cognitive decline at time of death (“control”, 26 participants). To determine which proteoforms may be associated with this categorical diagnosis, parallel linear models were fit with quantification ratio of each peptide as outcome variables, and diagnostic class, age at death, sex, post-mortem interval (hours) and number of years in education as explanatory variables. P-value adjustment was performed using Benjamini–Hochberg correction for multiple testing. All diagnostic class-related outcomes can be viewed in Supplementary Table 1.

171 peptides were associated with age at death, most of which increased in abundance with increasing age, including multiple peptides derived from CHGA/B and VGF. Peptides derived from cytoskeletal proteins also tended to increase in abundance with age, including proteoforms of dematin actin-binding protein (DMTN), paralemmin-1 (PALM) and microtubule-associated proteins 1/2 (MAP1/2), which may represent degradation of full-length cytoskeletal proteins in later life, leading to cytoskeletal instability^36^. Additionally, a large proportion of the proteoforms significantly associated with post-mortem interval were cytoskeletal-related. This is in concordance with past evidence noting significant degradation of full-length tubulin-α with increased post-mortem interval in human brain, as well as similar findings noted in other biological samples such as bone and skeletal muscle^37,38^.

257 peptides, derived from 142 proteins were significantly associated with sex, including CD99-derived cell adhesion peptides, which were enriched in males, a finding reliably observed across human brain and other tissue types^39,40^. Proteoforms derived from vesicle-associated membrane protein 2 (VAMP2) and VGF were also enriched in males vs females, two proteins which have recently shown secretory interplay^41^. Of the 348 peptides associated with education, many were cytoskeletal, with neurofilament light chain (NEFL) proteoforms making up 26 of the peptides significantly positively associated with increasing years in education.

### Differential enrichment of proteoforms by diagnostic class

**Figure 3:**
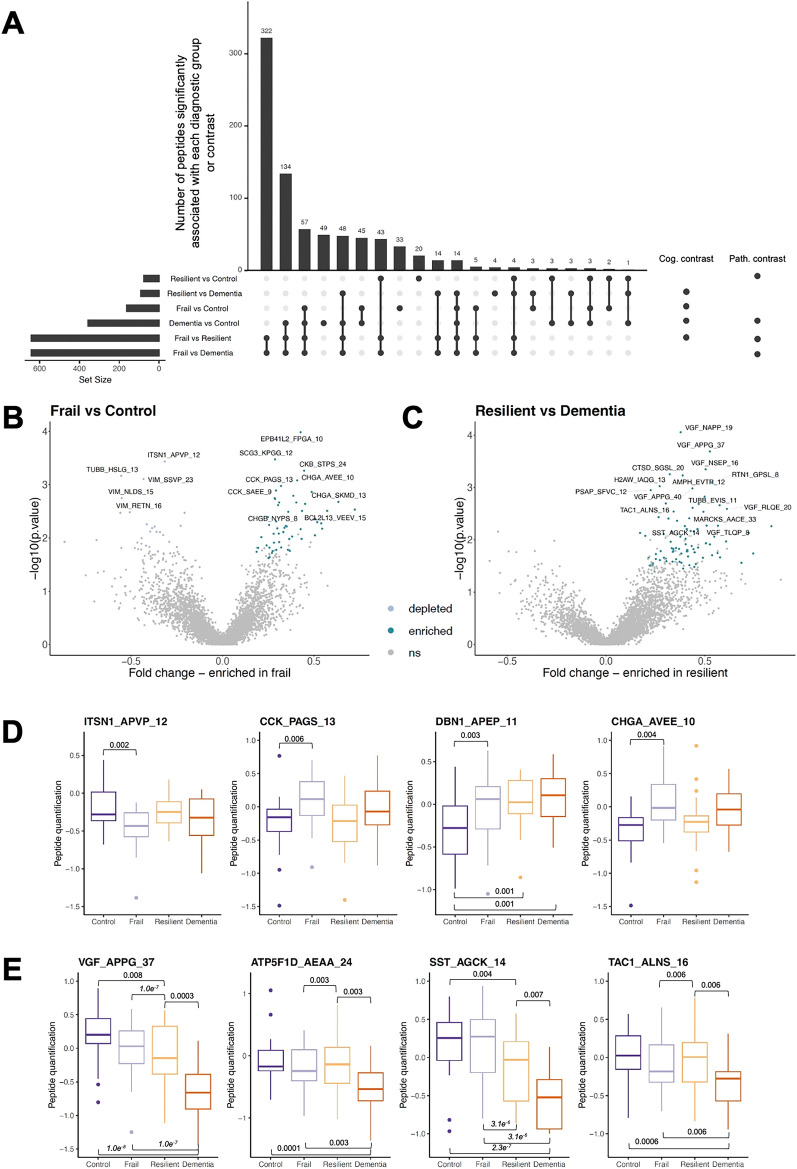
Results from categorical linear model. (**A**) UpSet plot of number of peptides significantly and uniquely associated with each diagnostic contrast. Cognitive (Cog.) contrasts represent situations in which the two compared diagnostic classes have opposite levels of cognition. Pathology (Path.) contrasts represent situations in which the two compared diagnostic classes have opposite levels of neuropathology. (**B**) Volcano plot of frail vs control contrast, showing differentially enriched peptides between groups. Enriched peptides (up) are differentially increased in frail samples in comparison to control samples, and depleted peptides (down) are differentially decreased in frail samples in comparison to control samples. The most significantly associated proteoforms are labelled. ns = not significant. (**C**) Volcano plot of resilient vs dementia contrast, showing differentially enriched peptides between groups. Enriched peptides (up) are differentially increased in resilient samples in comparison to dementia samples, and depleted peptides (down) are differentially decreased in resilient samples in comparison to dementia samples. The most significantly associated proteoforms are labelled. (**D**) Boxplots showing examples of four proteoforms that are differentially enriched between frail and control samples. Significant adjusted p-values are labelled. (**E**) Boxplots showing examples of four proteoforms that are differentially enriched between resilient and dementia samples. Significant adjusted p-values are labelled.

Of the 3696 modelled peptides, 807 unique proteoforms from 286 proteins were significantly associated with at least one diagnostic class contrast (Fig. 3A). To determine peptides linked to cognition status, the diagnostic class contrasts with disparate cognition outcomes but shared pathological signatures were considered—frail vs control (low pathology) and resilient vs dementia (high pathology). The peptides most significantly associated with the frail vs control contrast are depicted via volcano plot in Fig. 3B. The most significantly depleted proteoform in frail vs control brain was derived from intersectin-1 (ITSN1), a facilitator of clathrin-mediated endocytosis (Fig. 3B,D)^42^. Proteoforms of vimentin (VIM) also showed enrichment in control compared to frail samples. Notably, five CHGA-derived and six CHGB-derived proteoforms were significantly associated with this contrast, with all chromogranin-derived peptides showing enrichment in the frail group compared to controls (Fig. 3B,D). Secretogranin-3 (SCG3)-derived peptides also showed enrichment in frail brain, tentatively highlighting aberrant granin function with cognitive frailty. Five cholecystokinin (CCK) proteoforms were also reliably enriched in frail brain when compared to controls (Fig. 3B,D). Several cytoskeletal proteoforms were differentially enriched in frail brain, including an increase in abundance of multiple actin-binding Drebrin-1 (DBN1) peptides (Fig. 3D).

The proteoforms most significantly associated with the resilient vs dementia contrast are depicted via volcano plot in Fig. 3C. The majority of significant proteoforms were enriched in resilient brain compared to dementia. Several VGF proteoforms were enriched with cognitive resilience, including those previously shown to be depleted in AD (VGF_NAPP_19) and linked to resilience (VGF_APPG_37) (Fig. 3C,E)^19,43^. A previously defined peptide derived from protachykinin-1^44^ (TAC1), the gene which produces substance P and neurokinin A, and somatostatin (SST) were enriched in the resilient brain (Fig. 3C,E). Finally, mitochondrial-derived peptides were more abundant in resilient brain. (Fig. 3E).

### Differential enrichment of proteoforms by continuous cognitive measures

When stratifying the cohort into discrete diagnostic classes, arbitrary boundaries are set between low/high pathology/cognition. To determine which proteoforms may be associated with pathological and cognitive diagnosis on a continuous scale, avoiding subjective between-class limits, parallel linear models were fit with quantification ratio of each peptide as outcome variables, and global cognition Z-score, global pathology Z-score, age at death, sex, post-mortem interval (hours) and number of years in education as explanatory variables. P-value adjustment was performed using Benjamini–Hochberg correction for multiple testing. All cognition and pathology-related outcomes from such models can be viewed in Supplementary Table 2.

**Figure 4:**
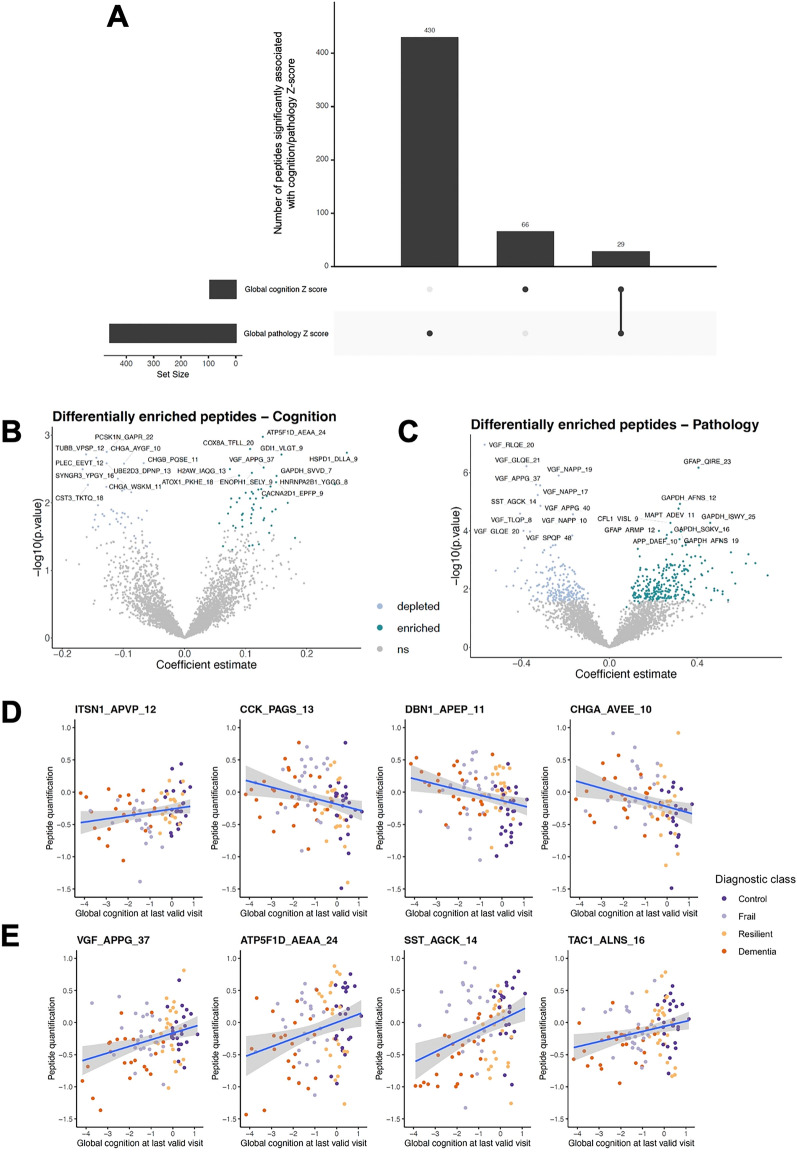
Results from continuous linear model. (**A**) UpSet plot of number of peptides significantly and uniquely associated with global measures of cognition or pathology, or both. (**B**) Volcano plot of differentially enriched peptides significantly associated with cognition Z-score. Peptides that were significantly associated with global cognition Z-score were plotted via volcano plot, with their coefficients on the x axis, and log transformed p-values on the y axis. Thus, peptides positively associated with increasing cognition score are shown to the right of x = 0. ns = not significant. **(C**) Volcano plot of differentially enriched peptides significantly associated with pathology Z-score. Peptides that were significantly associated with global pathology Z-score were plotted via volcano plot, with their coefficients on the x axis, and log transformed p-values on the y axis. Thus, peptides positively associated with increasing pathology score are shown to the right of x = 0. (**D**) Scatterplots showing examples of four proteoforms that were differentially enriched between frail and control samples. Correlations are shown with smoothed linear regression lines. (**E**) Scatterplots showing examples of four proteoforms differentially enriched between resilient and dementia samples. Correlations are shown with smoothed linear regression lines.

430 peptides from 196 proteins were significantly associated with global pathology, 66 (51 proteins) were associated with global cognition, and 29 (19 proteins) were linked to continuous measures of both pathology and cognition (Fig. 4A). Ten peptides from CHGA and five from CHGB were inversely associated with cognition, consistent with their enrichment in frail brain (Fig. 4B,D). The same trend is noted for proteoforms derived from CCK and DBN1 (Fig. 4D). Mitochondrial-derived peptides, including ATP synthase F1 subunit delta (ATP5F1D) and cytochrome c oxidase subunit 8A (COX8A) proteoforms, as well as VGF_APPG_37, were positively associated with cognition (Fig. 4B,E).

The majority of peptides significantly associated with global pathology were derived from VGF, with depleted VGF linked to increasing AD pathology (Fig. 4C). The canonical SST signalling peptide SST-14^45,46^, followed the same pattern. Proteoforms that increased in abundance with worsening neuropathology included, unsurprisingly, those derived from amyloid precursor protein (APP). The APP_DAEF_10 proteoform, which increased in abundance with worsening pathology, consists of the first ten residues of the Aβ42 peptide. Peptides of microtubule-associated protein tau (MAPT) and glial fibrillary acidic protein (GFAP) also showed increased levels with worsening pathology (Fig. 4C). Finally, several peptides from the glycolytic enzyme glyceraldehyde-3-phosphate dehydrogenase (GAPDH) were consistently enriched with pathology.

### Proteoforms associated with discrete and continuous measures of cognition display functional enrichment in specific biological pathways

**Figure 5:**
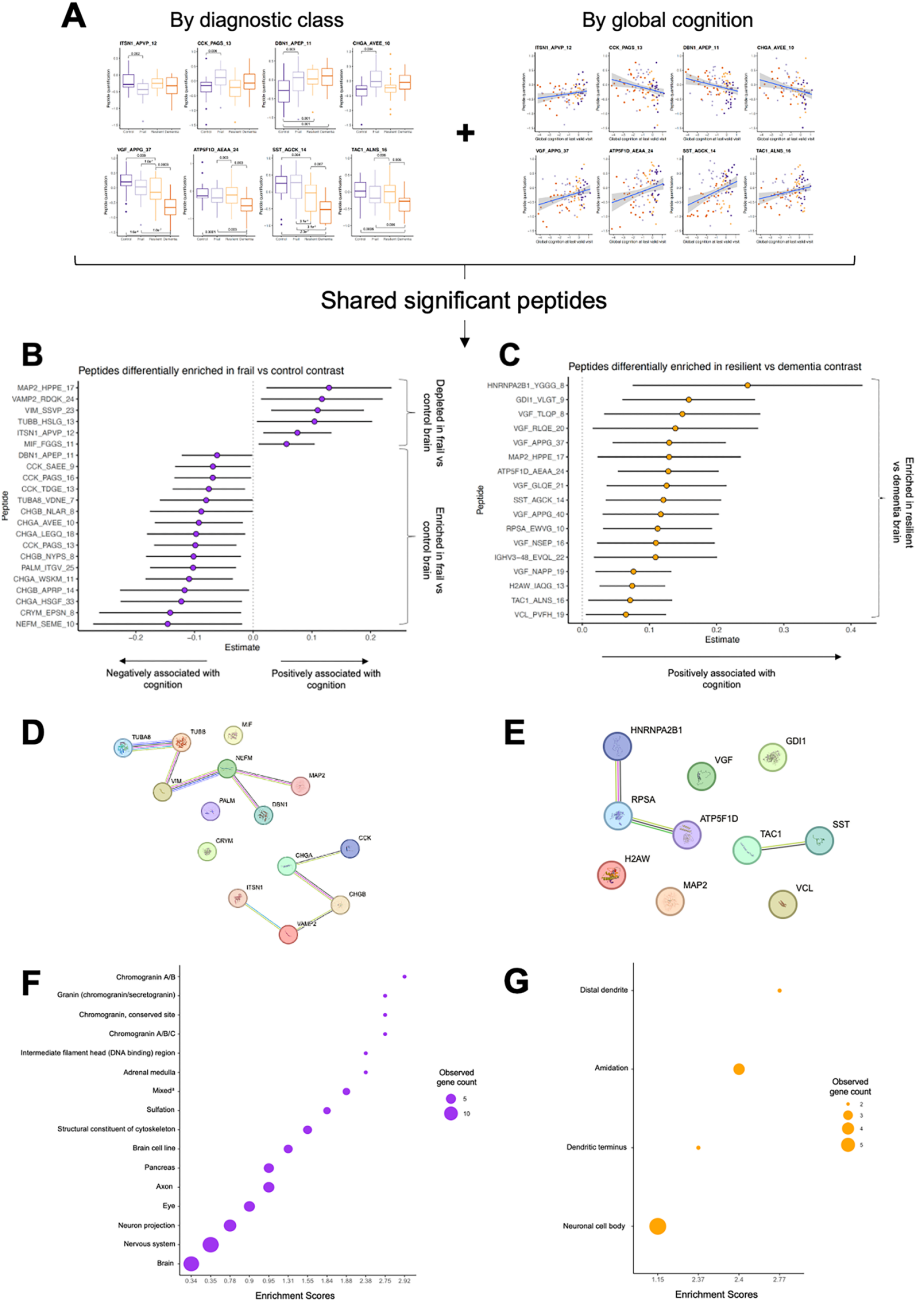
Merging categorical and continuous outcomes. (**A**) To discern which proteoforms were most robustly linked to cognition, outcomes from models including discrete diagnostic class and continuous global cognition Z-score were merged. Only peptides significantly associated with a cognitive contrast (frail vs control or resilient vs dementia) and associated with global cognition Z-score were included in further analysis. (**B**) 22 proteoforms were significantly associated with the frail vs control contrast and global cognition. Each row shows a proteoform plotted with its coefficient estimate from the continuous linear model*.* A positive coefficient represents proteoforms positively associated with cognition. Bars show 95% confidence intervals. (**C**) 17 proteoforms were significantly associated with the resilient vs dementia contrast and global cognition. Each row shows a proteoform plotted with its coefficient estimate from the continuous linear model. Peptides positively associated with cognition are enriched in resilient subjects. Bars show 95% confidence intervals. (**D**) STRING interaction network for the 14 genes that derive the 22 proteoforms differentially enriched between the frail and control cognitive contrast. (**E**) STRING interaction network for the 10 genes that derive the 17 proteoforms differentially enriched between the resilient and dementia cognitive contrast (without IGHV3-48 which was unknown by STRING). (**F**) A dotplot to show the enriched GO terms defined by STRING analysis of the gene network shown in (**D**) (frail vs control). ^a^The ‘Mixed’ term includes intermediate filament head, DNA-binding domain and distrobrevin. (**G**) A dotplot to show the enriched GO terms defined by STRING analysis of the gene network shown in (**E**) (resilient vs dementia).

To determine the most robustly cognition-associated proteoforms, the significant outcomes from the categorical and continuous models (Figs. 3 and 4), were merged (Fig. 5A). Using this approach, 39 peptides were consistently associated with cognition (Fig. 5B,C). 22 peptides, derived from 14 parent genes, were significantly associated with both the frail vs control contrast and continuous global cognition score. Six were depleted in the frail brain, including cytoskeletal proteoforms derived from MAP2, β-tubulin and vimentin, whilst 16 were increased with cognitive frailty, including CHGA/B, CCK and other cytoskeletal peptides such as α-tubulin and PALM (Fig. 5B). 17 peptides, from 11 parent genes, were significantly associated with both the resilient vs dementia contrast and continuous global cognition. All 17 proteoforms were increased in resilience, compared to dementia (Fig. 5C), with seven peptides derived from VGF. Proteoforms of SST and TAC1 were positively associated with cognitive resilience against AD. The same MAP2 proteoform (MAP2_HPPE_17) which exhibited depletion in frail brain was enriched with resilience.

To discern whether these enriched or depleted peptides (Fig. 5B,C) were functionally related, the STRING tool was used for gene ontology (GO) analysis^34^. A list of all proteins identified in brain by a recent large-scale human proteomic analysis was used as a background set (11,744 genes)^13^. GO enrichments within the frail vs control network included cytoskeletal constituents and granin family function (Fig. 5D,F) (Supplementary Table 3 shows GO enrichments for proteins significant in the frail vs control contrast across both models, Supplementary Fig. 1, Supplementary Table 4 show GO enrichments for proteins significant in the relevant categorical model outcomes only). Functional enrichments within the resilient vs dementia network included the distal dendrite, dendritic terminus and amidation (Fig. 5E,G) (Supplementary Table 5 shows GO enrichments for proteins significant in the resilient vs dementia contrast across both models, Supplementary Fig. 2, Supplementary Table 6 show GO enrichments for proteins significant in the relevant categorical model outcomes only).

### Pathways highlighted by GO analysis may be enriched in defined neuronal subpopulations

**Figure 6:**
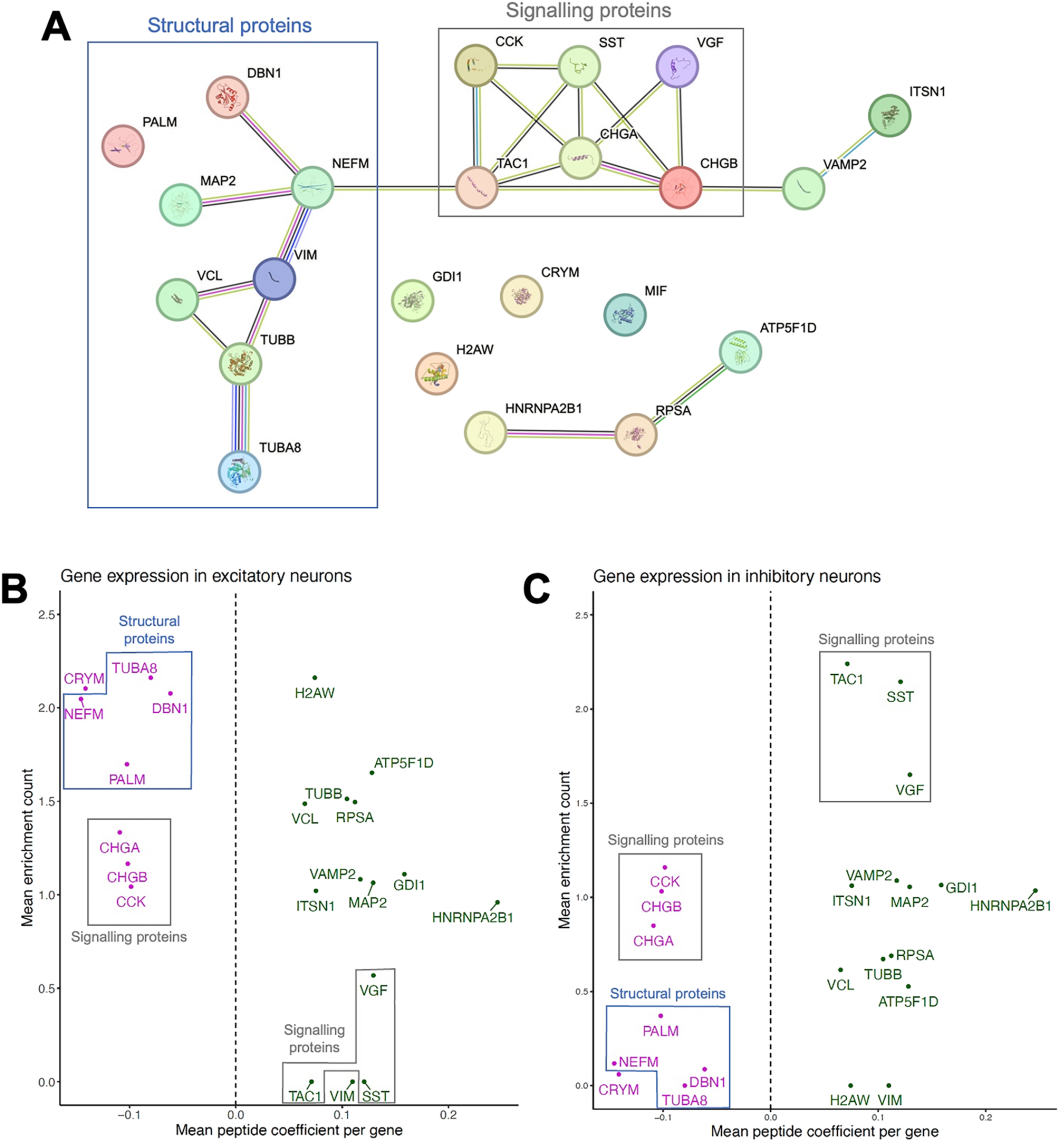
Functional clusters defined by STRING are enriched within specific neuronal subpopulations. (**A**) The 24 genes (without IGHV3-48 which was unknown by STRING) robustly associated with cognition were searched for interaction networks (medium confidence score, 0.400), against a background set of human brain genes from Johnson et al.^13^. Two main clusters of proteins were observed, signalling and structural proteins. (**B**) snRNA-seq data were integrated with cognition-associated genes. Mean expression count represents gene expression of excitatory neurons taken from Hodge et al.^35^. Mean peptide coefficient is the average linear model coefficient of peptides derived from the same gene, that were significantly associated with cognition. Genes to the left of x = 0 are negatively associated with cognition. For example, structural genes negatively associated with cognition are enriched across excitatory cell-types. (**C**) Mean expression count represents gene expression within inhibitory neurons taken from Hodge et al.^35^. Genes to the right of x = 0 are positively associated with cognition. For example, signalling genes positively associated with cognition are enriched in inhibitory neurons.

The 25 parent genes highlighted across both models (Fig. 5B,C) also underwent GO analysis together (Fig. 6A, Supplementary Table 7). This combined analysis highlighted two key biological clusters: structural proteins and signalling proteins (Fig. 6A). The structural cluster included proteins that hold cytoskeletal functions: DBN1, MAP2, neurofilament medium polypeptide (NEFM), VIM, vinculin (VCL), β-tubulin (TUBB), α-tubulin-8 (TUBA8) and PALM. A cluster of signalling proteins included members of the granin family (VGF, CHGA and CHGB), as well as the functionally related neuropeptides TAC1, SST and CCK. Functional connections between the chromogranin family, VAMP2 and ITSN1 were also highlighted (Fig. 6A), indicating the overlapping roles of these proteins in synaptic vesicle release and clathrin-mediated endocytosis.

To link differentially enriched peptides to particular cell types, single-nucleus RNA sequencing (snRNA-seq) data were obtained from a previous publication^35^. Mean enrichment scores of gene expression within grouped excitatory and inhibitory neuron classes were calculated and plotted against mean proteoform coefficients from the continuous linear models (Fig. 6B,C). Differences were seen in expression of genes of interest between excitatory and inhibitory neurons (Fig. 6B,C). Genes producing signalling peptides with positive cognition coefficients exhibited enrichment within inhibitory interneurons (INs), namely VGF, SST and TAC1 (Fig. 6C). Other members of the granin family that were negatively associated with cognition (CHGA, CHGB and CCK) did not show expression differences between neuronal types. Conversely, structural genes with negative cognition coefficients broadly exhibited enrichment within excitatory neurons, namely TUBA8, NEFM, DBN1 and PALM (Fig. 6B).

**Figure 7:**
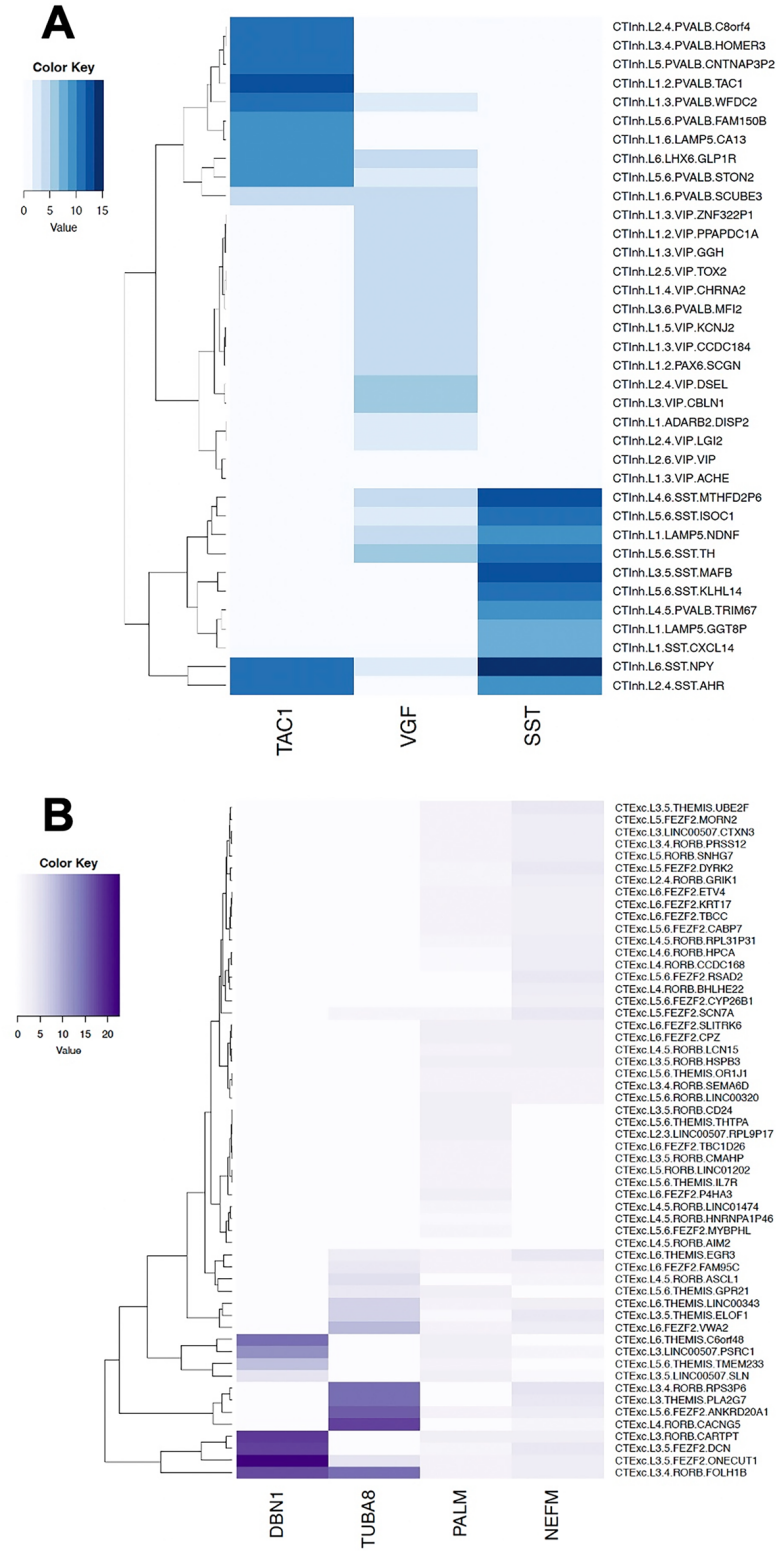
Genes of interest are expressed in specific A) inhibitory and B) excitatory cell-types. Single-nuclear sequencing data were taken from Hodge et al.^35^, and filtered for genes that were associated with cognition and enriched in either inhibitory or excitatory neurons overall: protachykinin-1 (TAC1), VGF (non-acronymic), somatostatin (SST), Drebrin-1 (DBN1), α-tubulin-8 (TUBA8), paralemmin-1 (PALM) and neurofilament medium polypeptide (NEFM). Heatmaps were constructed to show (**A**) the gene expression scores of signalling-related genes positively associated with cognition in inhibitory neuronal subtypes and (**B**) the gene expression scores of cytoskeletal-related genes negatively associated with cognition in excitatory neuronal subtypes. Of note, signalling-related genes associated with cognitive resilience showed enrichment in specific SST inhibitory interneuron subtypes (neuropeptide Y (NPY) SST INs) which have been highlighted recently as overrepresented in cognitively resilient individuals^14,20^ (**A**). Further, cytoskeletal-related genes associated with cognitive frailty showed enrichment in excitatory neurons, including RAR-related orphan receptor b (RORB) and THEMIS neurons, which have also been previously linked to cognitive manifestations of AD^20^ (**B**).

Due to their specific enrichment in inhibitory INs, we determined which cell-types SST, VGF and TAC1 were most strongly expressed in. Unsurprisingly, SST was most strongly enriched in SST INs, including layer 3/5 SST MAFB neurons, recently described as particularly vulnerable in AD^14^ (Fig. 7A). Layer 6 SST neuropeptide Y (NPY) INs were the only subtype to express all three IN-localised resilience-linked proteins, a subtype also highlighted by the same snRNA-seq study^14^. TAC1 was enriched in layer 1/2 parvalbumin (PVALB) TAC1 neurons, whilst VGF showed strongest enrichment in layer 2/3/4 vasoactive peptide (VIP) expressing neurons, as well as coexpression with SST in some SST IN populations. Across excitatory neuronal types, the excitatory-associated cytoskeletal genes (DBN1, TUBA8, PALM and NEFM) were co-enriched in layer 3/4 RAR-related orphan receptor b (RORB) and THEMIS-expressing excitatory neurons, both subclasses of intratelencephalic (IT) neurons (Fig. 7B). Recent transcriptomics has shown IT neuron retention with better residual cognition in AD subjects^20^.

## Discussion

Synaptic function has been highlighted as key in sustaining cognitive fitness in the face of AD-neuropathology, leading to detailed study into synaptic proteins of AD-dementia and AD-resilient brains^6,8,10–13^. However, a number of synaptic proteins function as peptide derivatives^47^, which complicates interpretation of quantification by classical tryptic mass spectrometry. Because semi-tryptic searches of whole-proteome tryptic data are limited by multiple testing concerns, we first experimentally enriched short proteins and peptides from human brain tissue by size filtration, before performing non-tryptic MS^19^. The main advantage of omitting trypsin use from MS sample preparation is that peptide ends are likely to arise from endogenous protease cleavage sites, thus increasing sensitivity for identification of relevant peptide proteoforms^19^. These nontryptic methods have disadvantages at the spectral identification stage, in that all residues must be considered as potential cleavage sites. The enrichment process during sample preparation means the resultant higher multiple testing during spectral identification is outweighed by the decreased complexity of the input sample, and the elimination of most full-length proteins from analysis^19^.

This study used nontryptic LC–MS to assess differential endogenous peptide abundance between 102 cortical samples from individuals within control, frail, resilient and dementia diagnostic classes. 39 proteoforms from 25 protein parents were robustly associated with cognitive state independent of pathology (Fig. 5B,C). The parent proteins of these proteoforms underwent GO analysis, to define functional interconnections between highlighted proteins. GO analysis broadly defined clusters of proteins involved in neuropeptide signalling (VGF, SST, TAC1) and cytoskeletal assembly (DBN1, TUBA8, PALM, NEFM) (Fig. 6A). Finally, publicly available snRNA-seq data^35^ were integrated with our data, showing that signalling-related proteoforms linked to cognitive resilience may localise to inhibitory SST-INs, whereas cytoskeletal proteoforms linked to frailty may be enriched in excitatory IT populations (Figs. 6 and 7). Taken together, our results suggest that differentially expressed proteoforms within neuropeptide signalling and cytoskeletal clusters may be important in the cognitive manifestations of AD, specifically within distinct cellular types.

Seven proteoforms of VGF (non-acronymic) were repeatedly significantly enriched in resilient vs dementia brain (Fig. 5C). VGF is a neurosecretory protein that primarily functions via its downstream proteoforms, with putative roles in metabolic regulation, Aβ clearance and synaptogenesis^48–50^. VGF has been reliably implicated in well powered multi-omic AD studies, with CSF^10,43,51,52^ and cortical^6,11–13,18^ depletion of VGF associated with AD and other dementia-types. Disparate biological roles have been defined for VGF peptides, including microglial modulation to aid Aβ clearance (VGF_TLQP_21) and enhanced synaptic plasticity through BDNF interaction (VGF_TLQP_62), yet the roles of many VGF peptides remain undefined, including the majority of those detected in the current study^48–50,53^. VGF_NAPP_19, which showed enrichment in resilient vs dementia brain, has previously been linked to energy homeostasis, with lower plasma NAPP_19 levels in high-fat diet-induced obese mice compared to control mice, and higher plasma NAPP_19 levels in slim compared to obese human euglycaemic subjects^54^. Given the growing interest in the connection between glucose regulation, diabetes and AD, this may be an important area for future study^55^. Though functional roles of the VGF peptides detected here remain to be established, consistent changes in VGF abundance across proteoforms between diagnostic classes in the present study, as well as previous literature, suggest clear roles of VGF peptides in cognitive functioning^15^. One counterintuitive finding in this study was that a selection of VGF proteoforms also increased with age, suggesting increased VGF release may be part of an ageing brain’s protective response to the negative effects of ageing.

A related peptide we identified as significantly associated with increased cognitive resilience is derived from somatostatin (SST_AGCK_14—generally known as SST-14). SST is an endocrine inhibitory hormone, with its brain derivative, SST-14, expressed throughout the cortex^56^. SST-14 mRNA is depleted in post-mortem AD cortex and hypothalamus, and decreased SST-14 brain abundance is documented with advanced ageing and declining cognition^57–59^. SST-14 immunoreactivity is widespread in neuritic Aβ plaques, and SST-deficient mice exhibit an Aβ load up to 1.5-times higher than wild-type mice^60,61^. Mechanistically, SST-14 may modulate expression of insulin-degrading enzyme to enhance Aβ proteolysis or prevent its aggregation^61–63^. Consequently, loss of SST expression may contribute to Aβ pathology accumulation. However, as we show that SST-14 levels are maintained despite Aβ burden in the AD-resilient vs AD-dementia brain, SST likely has roles in cognition beyond these putative interactions with Aβ. SST-14 binds strongly to proteins involved in synaptic vesicle maintenance and fusion, and is a known regulator of energy homeostasis, suggesting potential shared mechanisms with VGF_NAPP_19^46,64^. From integration of our data with snRNA-seq, it may be more likely that the changes we observe in SST-14 abundance peptide arise from changes in cell-type populations in the AD-cortex.

Cortical SST is primarily expressed in inhibitory INs which provide potent inhibition to neighbouring pyramidal cells, and have been designated amongst the strongest AD-associated cell type^14,20,65^. SST-expressing INs undergo selective degeneration in human AD cortex and rodent AD models, with SST neuron death linked to worsening cognitive decline^20,66,67^. Recently, subpopulations of SST INs, including neuropeptide Y (NPY) SST-INs, have been implicated in cognitive resilience, with overrepresentation of this cell-type in human cortex linked to resilience against AD neuropathology^14^. Accordingly, cerebrospinal fluid from individuals with AD-dementia has lower levels of NPY than aged-matched controls^68^. Notably, NPY SST-INs also express other neuropeptide signalling molecules that we associated with cognitive resilience, including protachykinin-1 (TAC1) and VGF (Fig. 7A). SST-INs sustain synaptic plasticity-dependent pyramidal cell activation, with SST loss correlated to pyramidal destabilisation and reduced motor learning in mice^69^. The relevance of GABAergic inhibitory neurons is furthered by the finding that enrichment of VGF in inhibitory, but not excitatory, neurons is associated with a significant delay in cognitive decline^15^.

**Figure 8:**
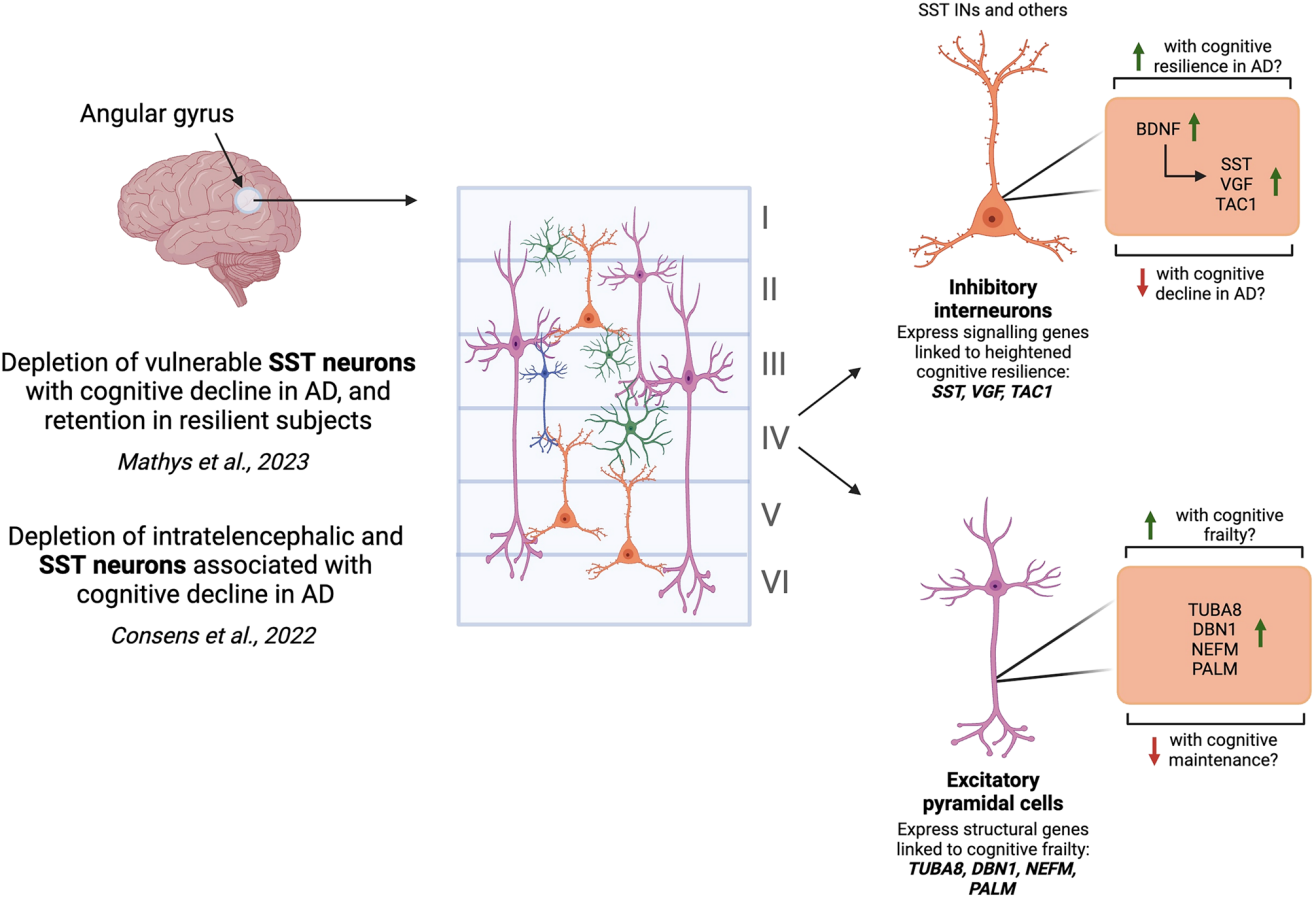
Somatostatin interneurons and specific excitatory neuronal subtypes may represent key cellular populations in mediating cognitive resilience and decline. Using previous single-nuclear sequencing data^35^, we found genes deriving proteoforms positively associated with cognition to be enriched in inhibitory interneurons, with SST NPY INs expressing all three genes in the cell signalling module (VGF, SST and TAC1). The expression of this module has been described as BDNF-dependent. SST INs should be considered in future study into cognitive resilience mechanisms^14,20^. The enrichment of structural-associated proteoforms, including those derived from TUBA8, DBN1, NEFM and PALM, was associated with cognitive decline. Previous study has shown greater abundance of intratelencephalic neurons, including RORB and THEMIS neurons, to be associated with slower cognitive decline^20^. The enrichment of cytoskeletal-associated proteoforms in these neurons in frail brain may represent neuronal degradation, and this should be studied further in the context of cognitive decline regardless of AD pathology. Created with BioRender.com.

An important link between SST, VGF and TAC1, resilience-associated neuropeptides that show increased parent gene expression in inhibitory INs, is BDNF (Fig. 8). These resilience-associated markers have been described as BDNF-dependent^70,71^. A highly powered study has shown higher *BDNF* expression to be associated with slower cognitive decline, even after controlling for the effect of neuropathology^17^. Further, BDNF is functionally linked with resilience through neuroprotective neuritin-1 (NRN1), which is known to facilitate dendritic resistance to Aβ pathology^11,12,72^. In the same exact tissue dissections, we have previously shown NRN1 to be upregulated in control synapses compared to AD-dementia synapses at the protein level, suggesting NRN1 may be an important driver of this functional protein module^11^.

While cognitive resilience is an emerging research domain, the mechanisms driving cognitive frailty (chronic cognitive decline in absence of measurable neuropathology) are less well described. Our GO analysis links secretory granule function to cognitive frailty, driven by CHGA and B (Fig. 5F). CHGA and B co-label with ~ 30% and ~ 15% of Aβ plaques respectively, as well as dystrophic neurites in AD cortex, implying their involvement in AD neuropathology^73–76^. In absence of Aβ, mimicking the frail environment, CHGA added to neuronal-microglial co-cultures induces microglial toxin release, provoking neuronal inflammation and apoptosis^77^. Since the chromogranins activate microglia in absence of AD-neuropathology, the immune environment of the frail brain should be studied further in future, to define differences that may underlie cognitive vulnerability^77,78^.

Cholecystokinin (CCK) is a peptide found in the gut and brain with widespread central effects^79^. Four CCK proteoforms were significantly enriched in the frail brain and negatively associated with cognitive Z-score (Fig. 5B). Mazurek and Beal showed decreased CCK expression in select cortical regions throughout the AD disease course, but stable levels in other cortical areas, highlighting a potential limitation of the focus on the angular gyrus in the current study^80^. Higher serum and brain CCK levels have previously been linked to better cognition in human and animal models^81^, which contrasts the CCK proteoform enrichment in frail brain we observe in the current study, as well as similar results from a previous proteomic analysis^11^. CCK and SST are synthesised by morphologically similar and locally projecting cortical INs^80^. CCK-expressing INs show a unique innervation pattern, including the disinhibition of nearby SST-INs, though SST-INs may not supply CCK-INs^82^. Thus, the overlap between SST and CCK-expressing interneurons, and their relevance to cognitive resilience and vulnerability, is an interesting future research topic.

In the present study, peptides derived from cytoskeletal proteins were also robustly linked to cognition. Seven of the 22 peptides associated with the frail vs control diagnostic contrast and cognitive decline were cytoskeleton-related, including proteoforms of MAP2, VIM, TUBB, DBN1, TUBA8, PALM and NEFM (Fig. 5B). Cytoskeletal function and remodelling lies at the centre of virtually all cellular processes including neuron-neuron communication, synapse formation and plasticity, and dendritic spine morphology. Dysfunction at the cytoskeletal level can therefore have widespread pathological impacts, and preservation of synaptic density is critically linked to AD resilience^8,83^. There are several possible explanations for the change in cytoskeletal peptide abundance with cognitive vulnerability. Cytoskeletal proteoforms enriched in frail brain, including DBN1, TUBA8, PALM and NEFM might represent degradation of full-length scaffolding proteins, indicating neuronal damage. Of note, these frail-enriched cytoskeletal proteins tend to be expressed in excitatory, rather than inhibitory, neurons (Fig. 6B). Previous transcriptomic work in ROSMAP samples has associated the retention of excitatory IT neurons with better residual cognition^20^. Our data show enrichment of frail-associated scaffolding genes in subsets of RAR-related orphan receptor b (RORB) and THEMIS-expressing ITs (Fig. 7B). RORB neurons may be subject to selective degeneration with advancing AD^84^, and ITs more generally have been highlighted to express neuroprotective genes underlying cognitive resilience against AD neuropathology^21^. Overall, parent genes of multiple cognition-associated proteoforms within signalling and scaffolding functional modules are enriched in cell-types with recurrent links to cognitive resilience and frailty—SST INs and IT neurons^14,20,21,58,84^. These populations should remain at the forefront of study into AD-related cognitive maintenance and decline.

### Study limitations

Firstly, despite the relatively large sample size and rich demographic data surrounding the cohort, the ROS cohort, from which most of these samples originate, consists of older Catholic nuns, monks and brothers from the United States only. This presents issues in data generalisability to the wider population, which should be addressed in future by the use of more diverse cohorts.

Secondly, the angular gyrus can exhibit unilateral connections to behaviour, with the left angular gyrus showing links to episodic simulation, and the right angular gyrus to location-related self-perception, as examples^85,86^. There may therefore be differences in protein and peptide expression between angular gyri from different sides of the brain. In addition, functional neuroimaging evidence suggests differences in angular gyrus activity between left-handed and right-handed individuals; suggesting that both the side of the brain the sample came from and the handedness of participants should be considered in future studies^87,88^.

Lastly, certain limitations of the proteomics methods exist, including the size of proteoform detected by MS. Proteoforms with previous links to cognition in AD may be too large for detection by the current MS protocol, for example VGF_TLQP_62. Thus, information drawn about larger peptides is limited in the current study. In terms of future directions, it may be interesting to use protease prediction databases to characterise which proteases might play active roles in cleavage of the relevant peptides. Since many proteases are immune activated, this information may offer further insight into the immune microenvironment of the resilient and frail brain.

### Supplementary Information

Supplementary Legends.

Supplementary Figure 1.

Supplementary Figure 2.

Supplementary Table 1.

Supplementary Table 2.

Supplementary Table 3.

Supplementary Table 4.

Supplementary Table 5.

Supplementary Table 6.

Supplementary Table 7.

## Data Availability

The PRIDE public repository details for the raw files for this work can be found at http://www.ebi.ac.uk/pride/archive/projects/PXD037367.
