# Supplementary material for: Interrogation of the human cortical peptidome uncovers cell-type specific signatures of cognitive resilience against Alzheimer’s disease
Source: Sci Rep. 2024 Mar 26;14:7161. doi: 10.1038/s41598-024-57104-z (PMC10966065; doi:10.1038/s41598-024-57104-z)
Supplement: Supplementary file 1 — Supplementary Legends. [file 41598_2024_57104_MOESM1_ESM.pdf]

Supplementary Figures Overview

Supplementary Table 1 – All categorical linear model outcomes

Supplementary Table 2 – All continuous linear model outcomes

Supplementary Table 3 – GO enrichments for proteins significant in frail vs control contrast across categorical and continuous linear models

Supplementary Table 4 – GO enrichments for proteins significant in frail vs control contrast in categorical linear models

Supplementary Table 5 – GO enrichments for proteins significant in resilient vs dementia contrast across categorical and continuous linear models

Supplementary Table 6 – GO enrichments for proteins significant in resilient vs dementia contrast in categorical linear models

Supplementary Table 7 – GO enrichments for proteins significant in frail vs control and resilient vs dementia contrast across both linear model types

Supplementary Figure 1 – STRING heatmap, all categorical outcomes (frail vs control)

Supplementary Figure 2 – STRING heatmap, all categorical outcomes (resilient vs dementia)

Supplementary Figure Legends

Supplementary Figure 1: Gene ontology analysis for frail vs control individuals. A) STRING interactions for the 46 genes that derive proteoforms significantly differentially enriched between frail and control individuals via linear modelling by diagnostic class (*Peptide quantification ratio ~ diagnostic class + age at death + sex + post-mortem interval + education years).* All significant STRING outcomes for this comparison can be found in **Supplementary Table 4**. **B)** Heatmaps show significantly enriched terms within the 'molecular function' and 'cellular components' gene ontology analysis outcomes. Mean peptide quantifications were calculated for each sample and plotted against significant STRING terms, to view which functional enrichments were represented by which diagnostic class (frail and control).

Supplementary Figure 2: Gene ontology analysis for resilient vs dementia individuals. A) STRING interactions for the 56 genes that derive proteoforms significantly differentially enriched between resilient and dementia individuals via linear modelling by diagnostic class (*Peptide quantification ratio ~ diagnostic class + age at death + sex + post-mortem interval + education years).* All significant STRING outcomes for this comparison can be found in **Supplementary Table 6**. Two of the 56 significant genes were not available to STRING and were not included in analysis (AQP-4 and IGHV3-48). **B)** Heatmaps show significantly enriched terms within the 'molecular function' and 'cellular components' gene ontology analysis outcomes. Mean peptide quantifications were calculated for each sample and plotted against significant STRING terms, to view which functional enrichments were represented by which diagnostic class (resilient and dementia).
