# Supplementary figures and images for: Interrogation of the human cortical peptidome uncovers cell-type specific signatures of cognitive resilience against Alzheimer’s disease
Source: Sci Rep. 2024 Mar 26;14:7161. doi: 10.1038/s41598-024-57104-z (PMC10966065; doi:10.1038/s41598-024-57104-z)

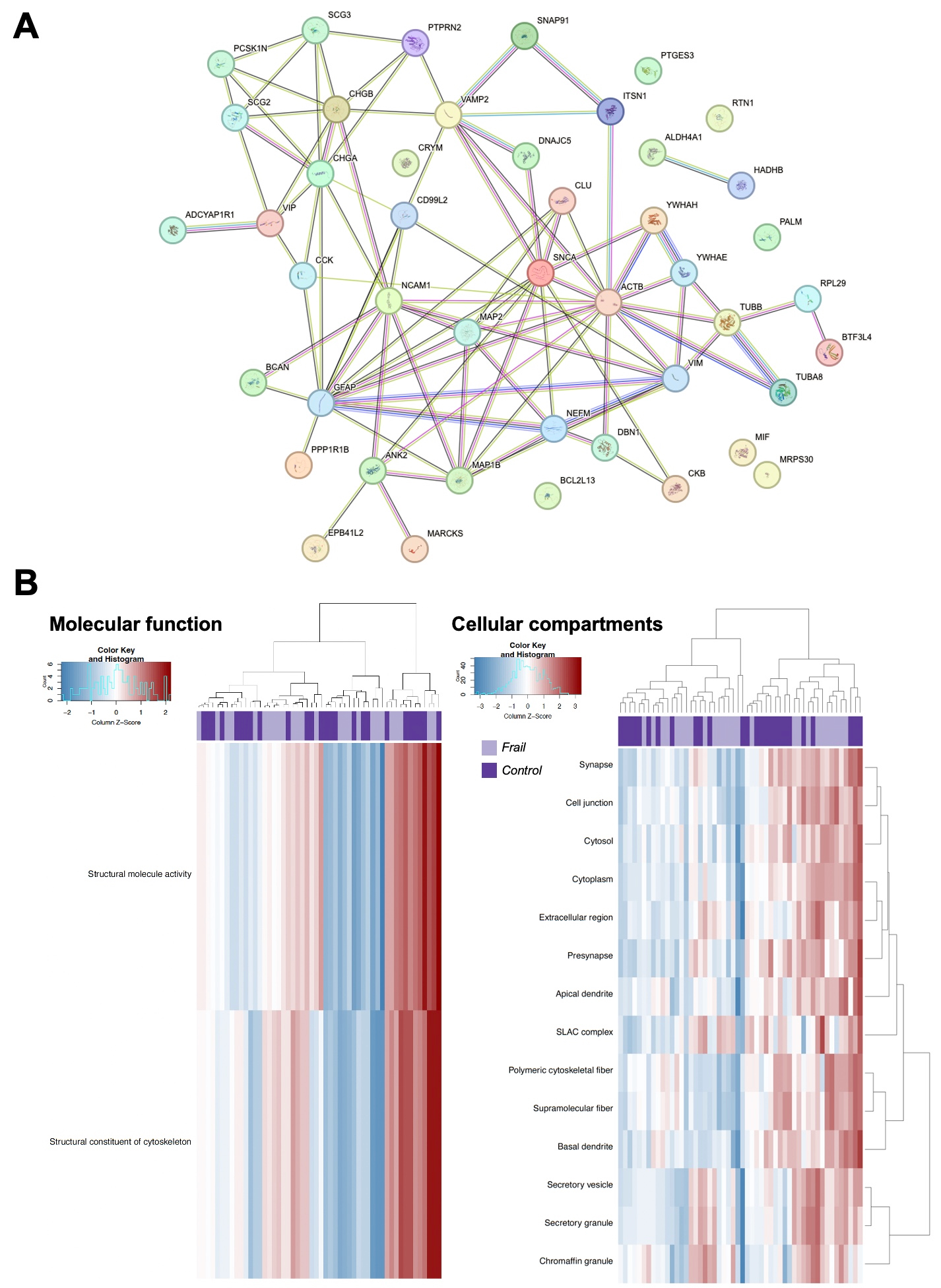

Supplement: Supplementary file 2 — Supplementary Figure 1. [file 41598_2024_57104_MOESM2_ESM.png]

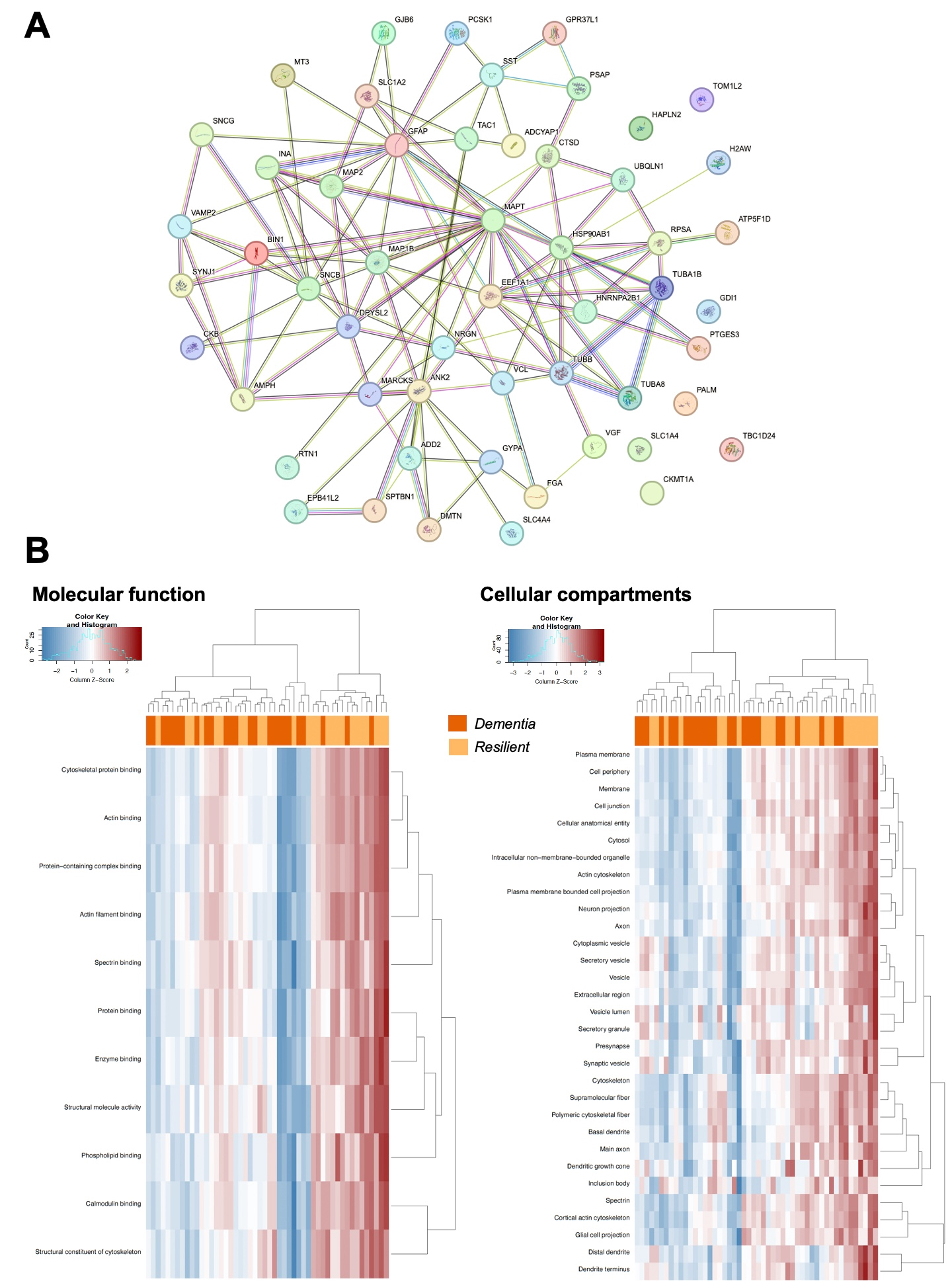

Supplement: Supplementary file 3 — Supplementary Figure 2. [file 41598_2024_57104_MOESM3_ESM.png]
